# Dissemination of Health-Related Research among Scientists in Three Countries: Access to Resources and Current Practices

**DOI:** 10.1155/2015/179156

**Published:** 2015-09-30

**Authors:** Rachel G. Tabak, Rodrigo S. Reis, Paul Wilson, Ross C. Brownson

**Affiliations:** ^1^Prevention Research Center in St. Louis, Brown School, Washington University in St. Louis, 621 Skinker Boulevard, St. Louis, MO 63130-4838, USA; ^2^Postgraduate Program in Urban Management, Pontifícia Universidade Católica do Paraná, 80.240-050 Curitiba, PR, Brazil; ^3^Department of Physical Education, Federal University of Parana, 80.240-050, Curitiba, PR, Brazil; ^4^Manchester Business School, University of Manchester, Manchester M15 6PB, UK; ^5^Division of Public Health Sciences and Alvin J. Siteman Cancer Center, Washington University School of Medicine, 621 Skinker Boulevard, St. Louis, MO 63130-4838, USA

## Abstract

*Objectives*. In public health and clinical settings insufficient dissemination of evidence-based practices limits the reach of new discoveries to broad populations. This study aimed to describe characteristics of the dissemination process by researchers across three countries (Brazil, United Kingdom, and United States), explore how designing for dissemination practices has been used, and analyze factors associated with dissemination. *Methods*. A similar online survey was used to query researchers across the three countries; data were pooled to draw cross-country conclusions. *Findings*. This study identified similarities and differences between countries. Importance of dissemination to nonresearcher audiences was widely recognized as important; however, traditional academic venues were the main dissemination method. Several factors were associated with self-rated dissemination effort in the pooled sample, but these predictive factors (e.g., support and resources for dissemination) had low prevalence. Less than one-third of researchers rated their level of effort for dissemination as excellent. Respondents reported limited support and resources to make it easier for researchers who might want to disseminate their findings. *Conclusion*. Though intentions show the importance of dissemination, researchers across countries lack supports to increase dissemination efforts. Additional resources and training in designing for dissemination along with improved partnerships could help bridge the research-practice gap.

## 1. Introduction

Though the United States (US), the United Kingdom (UK), and Brazil differ in many ways, across all three, there is significant spending on research to prevent and treat important health issues, such as cardiovascular disease [1–4]. While evidence is being produced, the pipeline to move this research into practice is far too long. It has been widely cited that it takes 17 years for research to make it into practice and that along the way most discoveries are lost, leaving only 14% of the evidence discovered to benefit health [5]. In both public health and clinical settings, there remains insufficient dissemination of evidence-based practices, resulting in new discoveries not reaching broad populations and populations most in need [6–8]. This is particularly true for cardiovascular disease prevention and treatment, for which many evidence-based strategies are available but have not had maximum population impact [9]. Dissemination has been defined as “an active approach of spreading evidence-based interventions to the target audience via determined channels using planned strategies” [10].

There is a need to speed up the pipeline from discovery to application (e.g., discovery of a new smoking cessation technique to widespread use across clinical and public health settings) [7]. Potential solutions to bring research to practice include involving stakeholders [11–16] in the research process (e.g., design, data gathering, and analysis) and/or evaluation process (also referred to as practice-based research [17]) and using theories and frameworks to guide dissemination efforts [18, 19]. Designing for dissemination (D4D) has been recommended as a strategy to help bridge the research-practice gap [20]. Researchers are encouraged to involve dissemination partners early on in the research process. Encouraging a role for potential adopters in research discovery creates a more collaborative approach [21–25]. Involving this future target audience in early, developmental phases of the research process, rather than at the end of a study [22, 26], can help researchers better incorporate issues related to external validity and translation for use in practice settings into intervention development [27–29].

The objectives of the current study are threefold. (1) Describe characteristics of the dissemination process by researchers across three countries; (2) explore how D4D practices have been used; (3) analyze factors associated with dissemination.

## 2. Methods

### 2.1. Sampling

Methods for sampling for each country have been presented in detail elsewhere [25, 30]. Briefly, in the US, sampling was conducted using the 12 journals with the highest impact factors in the category “public, environmental, and occupational health” using the lead author's affiliation, the NIH RePORTER database (an electronic tool for searching NIH-funded research projects), and researchers affiliated with the Prevention Research Centers (PRCs) Program of the Center for Disease Control and Prevention (CDC) from each PRCs website. These sources resulted in an initial pool of 488 valid investigators. Researchers were surveyed in 2012. In the UK, investigators from eight funding agencies conducting applied health services and public health research were identified. From these sources, the sample included 536 potential participants. Surveys took place in 2008. In Brazil a sample of 536 potential participants (all researchers classified as part of one of the health sciences areas as defined by Brazilian research agencies) was drawn from the database available at the Brazilian Council for Scientific and Technological Development or CNPq (Conselho Nacional de Desenvolvimento Científico e Tecnológico). Participants were surveyed from October to November 2012.

### 2.2. Questionnaire Development and Administration

Development of the UK survey [30] was informed by a systematic review of dissemination planning frameworks and strategies [18], which suggested key elements influencing the effectiveness of dissemination: planning activities, targeting audiences, selecting communication channels, and evaluating impact. Thus, the questionnaire had several parts. These were designed to elicit general researcher views and attitudes on the dissemination of research, think about a particular grant and capture any research impacts on health policy, clinical guideline development, or the organization and/or delivery of healthcare and services [31, 32], and obtain self-reported descriptions of research impacts (using four open-ended questions). This survey included 36 open and closed questions and took approximately 30 minutes to complete. Additional details about the UK survey can be found elsewhere [30]. This survey served as the basis for the surveys in the US and Brazil. The US and Brazil surveys included 35 items and 51 items and took respondents a median of 11 minutes and 15 minutes to complete, respectively. Only closed-ended items that were present on all three surveys were included in the current analysis. All surveys were conducted online; participants provided informed consent, and institutional review board approval was obtained from each of the three universities involved; additional details about the surveys and their administration can be found elsewhere [25, 30]. So that pooled analyses and those comparing across countries could be conducted, only survey items common across all three countries were included in the current analysis.

### 2.3. Data Analysis

Data were analyzed in SPSS version 20 (SPSS Inc, Chicago, IL). Distributions for each variable were explored across countries, and differences were explored using chi-squared tests. Associations between several predictor variables and self-rated effort to disseminate research findings to nonresearch audiences (i.e., “overall, how do you rate your efforts to disseminate your research findings to nonresearch audiences?” with response options as follows: excellent, good, adequate, poor, and not sure) were explored. The predictor variables included reasons why the researchers disseminate (including to influence policy/practice or to satisfy grant/contractual obligations), importance of dissemination, access to resources/structures (including a formal dissemination strategy or dedicated person for dissemination), adherence to designing for dissemination practices such as the stage at which dissemination-related activities occur, and dissemination processes and actions such as the frequency of producing research summaries. These predictors were selected as those hypothesized by the research team as most likely to be related to the outcome, and the set was limited to these variables to minimize the potential for extra comparisons and the likelihood of type 1 error. Multivariable logistic regression was used to determine odds ratios for excellent/good compared to poor self-rated dissemination effort for each country and in the pooled sample (with and without adjustment for country). The extreme categories were created to maximize differences between groups.

## 3. Results

Across countries, the samples included similar number of researchers with 277 respondents in Brazil, 232 in the UK, and 266 in the US. Response rates were 42% in Brazil and around half for the UK (50%) and the US (54%).

### 3.1. Reasons to Disseminate

Across countries, researchers cited similar reasons to disseminate their findings (Table 1). For the pooled data, roughly eight out of ten respondents considered “to raise awareness of the findings,” “to influence policy or practice,” and “to influence practice” as the main reasons to disseminate their findings. Some differences existed between countries. Stimulating discussion or debate (86%) and justifying public funding (60%) were reported most frequently by Brazilian researchers compared to researchers in the other countries, while satisfying grant/contractual obligations (15%) was lower among these researchers. UK respondents ranked influencing policy, attracting future funding (62%), and raising the organizational profile (65%) as higher than the other two countries, while they reported promoting public understanding of science (40%) as lower. Compared to researchers in the other countries, US researchers selected promoting public understanding of science (58%) and to satisfy grant/contractual obligations (41%) frequently. However, US researchers selected the reasons of justifying public funding (39%) and raising the organizational profile (42%) less often than researchers in other countries.

### 3.2. Importance

In the pooled sample, almost two-thirds of respondents reported that dissemination to nonresearch audiences was very important to their own research and roughly half of the participants reported that dissemination was very important for their unit/department. The importance of dissemination differed by country (Table 1). Seventy-eight percent of Brazilian researchers and 70% of UK researchers reported that dissemination of their own research was very important, but this was the case for 54% of US researchers. Though the number reporting importance of dissemination of their unit/department's work was slightly lower overall (59% versus 66% for their own work), the pattern by country remained, with 76% of Brazilian and 66% of UK, but only 38% of US, researchers reporting this was very important.

### 3.3. Resources/Structures

Access to a formal dissemination strategy (27%) and a dedicated person or team (36%) was low in the pooled sample (Table 1). However, compared to Brazil and UK researchers, US researchers reported the highest access to resources (53%). In Brazil and UK roughly a 30% and 20% reported access to a formal communication strategy/plan or a dedicated person, respectively, while one-third and one-half of US participants reported access to these resources.

### 3.4. Dissemination Processes and Actions

Table 1 also presents the methods researchers use for this dissemination by country and for the total sample. Across countries, the most frequently reported methods were academic journals (99% overall) then academic conferences (81% overall). Methods differed significantly by country. Brazil did not rank any of the methods significantly more frequently than any other countries but ranked press releases (33%), other conferences (22%), newsletters (14%), and targeted mailings (3%) less frequently. Researchers from the UK reported to funders (91%) and other conferences (55%) more frequently than other countries; they reported face-to-face meetings (40%) less often. The only method US researchers reported least frequently was seminars and/or workshops (61%). US researchers reported several methods more frequently than other countries; these included press releases (62%), media interviews (51%), newsletters (45%), and email alerts (22%).

### 3.5. Designing for Dissemination

Table 1 reveals that, in the overall sample, less than one-half of researchers reported planning dissemination-related activities early and roughly one-third reported they always or usually produce summaries for nonresearch audiences. Additionally, several important actions related to designing for dissemination and other dissemination-related activities showed differences across countries (Table 1). Brazilian researches most frequently reported planning dissemination-related activities early (60%, compared to 45% in the US and 35% in the UK). However, Brazilian researches most frequently reported rarely or never producing summaries for nonresearch audiences (42% compared to 30% in the US and 20% in the UK). Though the frequency across all countries was low (17%), 22% of Brazilian researchers reported that they always or usually evaluate the impact of their research on changing public health practice or policy; 26% of US researches reported they never do this. Use of frameworks was also quite low across all countries (12% reporting always or usually), though it was the lowest in the UK (9%) and the highest in the US (17%).

**Figure 1:**
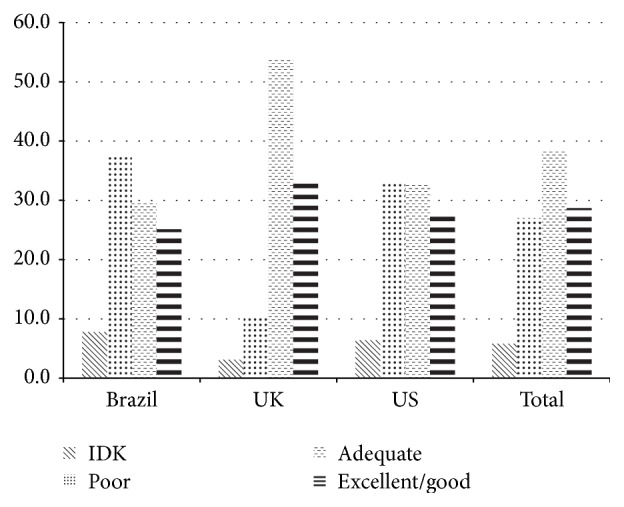
Self-rated dissemination effort to disseminate public health findings to nonresearch audiences in Brazil, US, and UK % (IDK = I do not know).

Overall, 18% of researchers reported spending greater than 20% of their time on dissemination-related activities (Table 1), with the highest in Brazil (28%) and the lowest in the US (11%). Finally, roughly one-third of the participants reported their own effort to disseminate their findings as excellent or good, though the UK had the highest percentage (33%) compared to Brazil (25%) and the US (28%) (Figure 1).

### 3.6. Factors Associated with Dissemination Efforts

Several factors were associated with self-reported dissemination effort, with important differences across countries (Table 2). Researchers reporting that they disseminate their findings to influence policy or practice were more likely to self-rate themselves as putting more effort toward dissemination (OR = 3.8; 95% CI = 1.7–8.8); this was not significant for the UK or Brazil. Interestingly, there was no relationship between disseminating to satisfy grant/contractual obligations and effort to disseminate; indicating intention to disseminate may not translate to behavior. Those reporting a unit/department/school with formal communication/dissemination strategy had higher odds of better self-rated effort in the pooled data (OR = 2.9; 95% CI = 1.8–4.7) as well as all individual countries except the US. However, the positive association between reporting a dedicated person/team and self-reported effort to disseminate was only significant in the pooled analysis (adjusted for country) (OR = 1.6; 95% CI = 1.0–2.5). In the pooled sample (OR = 8.2; 95% CI = 5.8–11.7) as well as all the individual countries, reporting producing research summaries for nonresearch audiences was also associated with higher self-rated effort to disseminate. Finally, researchers who planned dissemination at an earlier stage of their research process (pooled sample) were more likely to report excellent/good dissemination efforts (OR = 3.3; 95% CI = 2.3–4.7).

## 4. Discussion 

This exploration of the characteristics of the dissemination process by researchers across three countries found a number of similarities as well as important differences between countries. Researchers seem to recognize that dissemination of their findings to nonresearch audiences is a main reason for their research and rate it as important; however, the main method they use to reach this audience is through traditional academic venues. Overall, most of the variables explored were associated with self-rated dissemination effort in the pooled sample. However many of these factors had low prevalence in the countries explored. It seems there are limited support and resources to make it easier for researchers who might want to disseminate their findings. The study also revealed that less than one-third of researchers felt the level of effort they put toward dissemination efforts was excellent. This may in part explain the speed at which research is being translated into practice [7, 33]; many researchers may lack access or may be unaware of the effective methods of knowledge translation.

Our modeling indicated factors related to increased success in dissemination by researchers, and these included access to the right resources and practicing designing for dissemination activities. Researchers reporting a unit/department/school with a formal communication dissemination strategy had higher odds of better self-rated effort. Unfortunately, even though access was somewhat higher in the US, it was low across the three countries (with only about one-third of participants in the pooled sample reporting some access), indicating a lack of resources for dissemination in developed and developing countries. Thus our findings point to the potential for provision of such resources to enhance the ability of researchers to improve their efforts to disseminate. Alternately, formation of a transdisciplinary team may be a more feasible approach. It may not be feasible or even advisable for a public health researcher to develop all of the necessary dissemination skills. Teaming with a communications or marketing expert might allow for optimal dissemination. Whether the dissemination efforts are by the researchers themselves, team members with dissemination expertise, or outside experts, they will likely involve some type of technology [34–36]. Many of the methods of dissemination mentioned by researchers in the current study are conducted at least in part online. In particular, the growth of online publishing and potential of social media now offers a multitude of low cost, high reach channels for the dissemination of information about prevention and treatment of cardiovascular disease. Future efforts should explore how to maximize the multitude of technological platforms and utilize target audience preferences for effective dissemination [37].

Better connections between researchers and practice are another avenue which has been suggested as a way to improve translation of research to practice [38, 39]. This may be particularly true in low and middle income countries [40]. This analysis found significant associations between whether the researcher had practice-based experience and self-rated dissemination effort in two of the countries explored (data not shown) [25]. Training programs offering researchers practice-based experience and also valuing practice experience in faculty recruitment and promotion requirements may serve as a way to build these connections. For example, the American Heart Association conducts a course: Seminar on the Epidemiology and Prevention of Cardiovascular Disease that brings together researchers and practitioners working on cardiovascular health promotion [41]. Such programs could be expanded to include management and treatment of cardiovascular disease, furthering the impact of research into practice.

Designing for dissemination is an active process that helps to ensure that public health interventions, often evaluated by researchers, are developed in ways that match well with adopters' needs, assets, and time frames [25]. Additional training in designing for dissemination may be needed across countries for researchers developing cardiovascular disease prevention and treatment interventions [42]. Structural factors in the architecture of research institutions may also be important barriers to engage in designing for dissemination [43]. There was a difference in both the importance of dissemination and the use of designing for dissemination practices among Brazilian researchers; further, the US lagged the other countries. Though it was not universal across activities, Brazilian researches most frequently reported planning dissemination-related activities early, always or usually evaluating the impact of their research on changing public health practice or policy, and spending greater than 20% of their time on dissemination-related activities. These factors may be related to the fact that researchers embracing the importance of dissemination may also be the researchers who are more likely to engage in designing for dissemination activities, such as involving stakeholders earlier in the research process. Some cardiovascular disease prevention programs, which were designed with dissemination in mind, have been scaled up, allowing them to have broad, population health impacts [9]. For example, the Child and Adolescent Trial for Cardiovascular Health (CATCH), an evidence-based program for youth in United States schools, has engaged diverse stakeholders and has been widely disseminated and adopted [44–46]. In Brazil, Guide for Useful Interventions for Activity in Brazil and Latin America (GUIA), a cross-national academic-government partnership, used evidence of effectiveness to trigger political action to scale up Academia da Saúde (a community-based physical activity intervention) at the national level [47].

While the top reasons to disseminate were similar across countries, there were apparent differences observed, which indicate that messaging to enhance researcher dissemination may need to be different across countries, as the reasons behind such efforts differ. This may reflect local culture, political environment, and/or other factors. For example, in the UK applied health research has a strong emphasis (from the major funding source National Institute for Health Research) on producing research to address National Health Service priorities and to support decision making by health professionals and policy makers, which may have led to more frequent reporting of influencing policy and attracting future funding among this population. However, relative to the top reasons for dissemination, these were reported less frequently. The role of funders may therefore be important not only because many dissemination activities are often unfunded [48] but also because funders can set the priority of the project to include dissemination [49–51]. Funders of research in the prevention, management, and treatment of cardiovascular disease may consider making this an important priority in the development of calls for proposals and review criteria. Repeating this survey in other countries might identify additional points for country-specific actions. There are additional benefits to repeating the survey in these three countries and replicating this project in other countries so they might identify areas for improvement. This might allow countries to see how they can improve dissemination to nonresearch audiences, as many countries are spending large amounts of money on research. Further, a coordinated approach to administration of surveys similar to the ones used in the current work in a large number of countries, with a variety of education and funding structures, would allow for additional pooled analyses. This might shed light on whether there are systematic differences in the reasons researchers disseminate their findings as well as what structures might be most supportive.

This study had limitations worth noting. First, each country conducted the study in a different context; thus common demographic measures are not available across countries, and there was variation in time of delivery. Considering the speed at which the field of dissemination research is moving, it is possible that substantive changes may have occurred in the use of research dissemination practices. The differences in demographics collected across countries prevent presentation of field of research and publication records for the survey respondents. However, this study did not aim to look at individual-level predictors of dissemination. Further, questions may have been interpreted differently across countries, perhaps leading to some of the cross-country differences observed. Additionally, the researchers in the study were not a systematic nationwide sample, but they were meant to represent sectors of research. The administration of the survey in only three countries limits the generalizability of the findings, though the countries included were diverse in context. As this data was collected by self-report, there is always the potential for social desirability bias. Further, information on who did and did not participate is not available, leaving open the potential for bias, as the sample likely includes those most interested. Finally, the cross-sectional nature of the study limits our ability to determine causality. Despite these limitations, this study provides a first of its kind look at dissemination and dissemination-related factors among researchers in health-related fields across three countries.

## 5. Conclusions

This study identified that researchers are interested in disseminating the results of their research to raise awareness and to influence practice and/or policy. Though intentions seem to show the importance of dissemination, researchers across countries appear to lack supports to enhance their dissemination efforts. Additional resources as well as training in designing for dissemination along with improved partnerships could help bridge the research-practice gap.

## Figures and Tables

**Table 1:** Reasons, resources, and methods used to disseminate the results of research in public health in Brazil, US, and UK %^a^ (*n*).

Dissemination characteristics	Brazil	United Kingdom	United States	Total	*P* value (*χ* ^2^)
Reasons to disseminate^b^					
Raise awareness of the findings	92.1 (209)	93.1 (216)	94.4 (251)	93.2 (676)	.598
Influence practice or policy	91.2 (207)	93.1 (216)	90.2 (240)	91.4 (663)	.512
Influence practice	81.1 (184)	83.6 (194)	80.1 (213)	81.5 (591)	.583
Influence policy	78.0 (177)	85.3 (198)	75.6 (201)	79.4 (576)	.021
Stimulate discussion or debate	86.3 (196)	74.6 (173)	70.7 (188)	76.8 (557)	<.01
Attract future funding	52.4 (119)	62.5 (145)	52.3 (139)	55.6 (403)	.037
Raise the organizational profile	52.4 (119)	64.7 (150)	42.1 (112)	52.6 (381)	<.01
Promote public understanding of science	47.1 (107)	40.1 (93)	57.9 (154)	48.8 (354)	<.01
Justify public funding	60.4 (137)	48.3 (112)	39.1 (104)	48.7 (353)	<.01
Satisfy grant/contractual obligations	15.0 (34)	36.6 (85)	41.4 (110)	31.6 (229)	<.01
Improve your own communication	29.1 (66)	24.1 (56)	21.1 (56)	24.6 (178)	.117

Importance of dissemination to nonresearch audiences					
Importance to your own work					
*Very important *	77.7 (171)	69.6 (160)	54.2 (143)	66.4 (474)	
*Important *	19.5 (43)	24.3 (56)	24.2 (64)	22.8 (163)	
*Somewhat/not important/NS *	2.7 (6)	6.1 (14)	21.6 (57)	10.8 (77)	<.01
Importance to the work of your unit/department					
*Very important *	75.9 (167)	65.7 (151)	37.7 (100)	58.5 (418)	
*Important *	22.7 (50)	26.5 (61)	24.5 (65)	24.6 (176)	
*Somewhat/not important/IDK* ^c^	1.4 (3)	7.8 (18)	37.7 (100)	16.9 (121)	<.01

Resources & structures for dissemination in unit/department					
Formal communication/dissemination strategy or plan	27.8 (62)	20.2 (46)	31.8 (84)	26.9 (192)	.014
Dedicated person or team responsible for dissemination-related activities	32.0 (71)	20.6 (47)	52.6 (140)	36.0 (258)	<.01

Method of dissemination^b^					
Academic journals	98.7 (224)	97.8 (227)	100.0 (266)	98.9 (717)	.067
Academic conferences	91.6 (208)	96.1 (223)	92.5 (246)	93.4 (677)	.117
Report to funders	79.7 (181)	91.4 (212)	68.0 (181)	79.2 (574)	<.01
Seminars and/or workshops	69.2 (157)	71.1 (165)	60.9 (162)	66.8 (484)	.035
Press releases	32.6 (74)	48.3 (112)	62.0 (165)	48.4 (351)	<.01
Face to face meetings	49.8 (113)	40.1 (93)	53.4 (142)	48.0 (348)	.01
Other conferences	21.6 (49)	55.2 (128)	42.5 (113)	40.0 (290)	<.01
Media interviews	32.6 (74)	31.9 (74)	50.8 (135)	39.0 (283)	<.01
Newsletters	13.7 (31)	39.2 (91)	45.1 (120)	33.4 (242)	<.01
Email alerts	6.2 (14)	7.8 (18)	22.2 (59)	12.6 (91)	<.01
Targeted mailings	3.1 (7)	16.4 (38)	16.2 (43)	12.1 (88)	<.01

Designing for dissemination/processes/actions					
Stage in the research process that dissemination-related activities are planned					
*Early *	60.0 (132)	35.2 (80)	45.1 (120)	46.6 (332)	
*Late *	36.8 (81)	64.8 (147)	39.1 (104)	46.6 (332)	
*Never *	3.2 (7)	0.0 (0)	15.8 (42)	6.9 (49)	<.01
Frequency that research summaries/key messages are written for specific nonresearch audiences					
*Always/usually *	34.7 (76)	31.3 (71)	32.0 (85)	32.6 (232)	
*Sometimes *	22.8 (50)	48.5 (110)	37.6 (100)	36.5 (260)	
*Rarely/never/NS *	42.5 (93)	20.3 (46)	30.5 (81)	30.9 (220)	<.01
Frequency the impact of your research is evaluated?					
*Always/usually *	22.4 (49)	13.3 (30)	15.5 (41)	16.9 (120)	
*Sometimes/rarely *	55.7 (122)	69.5 (157)	56.8 (150)	60.5 (429)	
*Never *	19.2 (42)	17.3 (39)	26.5 (70)	21.3 (151)	
*Not sure *	2.7 (6)	0.0 (0)	1.1 (3)	1.3 (9)	<.01
Frequency that a framework/theory is used to plan dissemination-related activities					
*Always/usually *	10.8 (24)	8.8 (20)	16.7 (44)	12.3 (88)	
*Sometimes/rarely *	22.4 (50)	48.5 (110)	38.4 (101)	36.6 (261)	
*Never *	60.1 (134)	39.6 (90)	25.9 (68)	41.0 (292)	
*Not sure *	6.7 (15)	3.1 (7)	8.7 (23)	6.3 (45)	
*Do not plan dissemination activities *	0.0 (0)	0.0 (0)	10.3 (27)	3.8 (27)	<.01
Time dedicated to dissemination-related activities to nonresearch audiences.					
<5%	36.5 (80)	31.4 (71)	54.4 (143)	41.5 (294)	
5–20%	35.6 (78)	52.7 (119)	34.6 (91)	40.7 (288)	
>20%	27.9 (61)	15.9 (36)	11.0 (29)	17.8 (126)	<.01

^a^% within local; ^b^those responding “yes”; ^c^IDK = I do not know.

**Table 2:** Regression of predictor variables on self-rated dissemination^a^ effort separately for each country and pooled (unadjusted and adjusted for country) OR (95% CI).

Dissemination characteristics	Brazil	United Kingdom	United States	Pooled (crude)	Pooled (adj^b^)
*Reasons *					
Influence policy OR to influence practice	2.1 (0.5–8.3)	5.5 (0.9–35.0)	**4.5** (**1.2**–**16.5**)	**3.8 (1.7**–**8.7)**	**3.8 (1.7**–**8.8)**
To satisfy grant/contractual obligations	0.5 (0.2–1.3)	1.1 (0.4–3.0)	0.9 (0.5–1.7)	0.7 (0.5–1.2)	0.8 (0.5–1.3)
*Resources *					
Unit/department/school has formal communication/dissemination strategy	**3.0 (1.4**–**6.5)**	—^c^	1.8 (0.9–3.6)	**2.4 (1.5**–**3.9)**	**2.9 (1.8**–**4.7)**
Dedicated person/team for dissemination in unit/organization	1.7 (0.8–3.5)	2.6 (0.7–9.6)	1.3 (0.7–2.4)	1.2 (0.8–1.9)	**1.6 (1.0**–**2.5)**
*Methods/actions *					
Frequency that research summaries/key messages are written for specific nonresearch audiences	**5.9 (3.5**–**9.9)**	**4.8** (**2.2**–**10.1**)	**21.9** (**10.7**–**44.9**)	**8.5 (6.0**–**12.0)**	**8.2 (5.8**–**11.7)**
Stage in the research process when planning dissemination-related activities	**3.2 (1.5**–**6.7)**	3.2 (0.98–10.2)	**3.9** (**2.4**–**6.6**)	**2.8 (2.0**–**4.0)**	**3.3 (2.3**–**4.7)**

^a^The reference category is poor (versus excellent/good); ^b^adjusted for country w/dummy variables; ^c^could not be estimated due to small cell sizes.
